# Supplementary material for: Transcriptomic Analysis of Human Skeletal Muscle in Response to Aerobic Exercise and Protein Intake
Source: Nutrients. 2023 Aug 7;15(15):3485. doi: 10.3390/nu15153485 (PMC10421363; doi:10.3390/nu15153485)
Supplement: Supplementary file 1 [file nutrients-15-03485-s001.zip › Supplementary+Figures-0804.pdf]

The description of supplementary Tables

Supplementary Table S1 The summarization of top 5 up- and down-regulated DEGs, co-DEGs ranking by the absolute value of log2FC and gene expression network of skeletal muscle in response to different interven-tions within each dataset

Supplementary Table S2 The hub gene screening from co-DEGs calculated by Cytohubba plugin in Cytoscape software (ranked by Degree value).

Supplementary Table S3 The GO enrichment analysis of co-DEGs from varied interventions in different populations.

Supplementary Table S4 The KEGG enrichment analysis of co-DEGs from varied interventions in different populations.

Supplementary Table S5 The gene sets related GO terms from GSEA in each dataset by different interventions.

Supplementary Table S6 The gene sets related KEGG pathways from GSEA in each dataset by different interventions.

Note: YM, the younger male; OM, the older male; OF; the older female; AE, aerobic exercise; DEGs, differentially expressed genes; co-DEGs, co-differentially expressed genes; GO, Gene Ontology; KEGG, Kyoto Encyclopedia of Genes and Genomes; GSEA, gene set enrichment analysis.

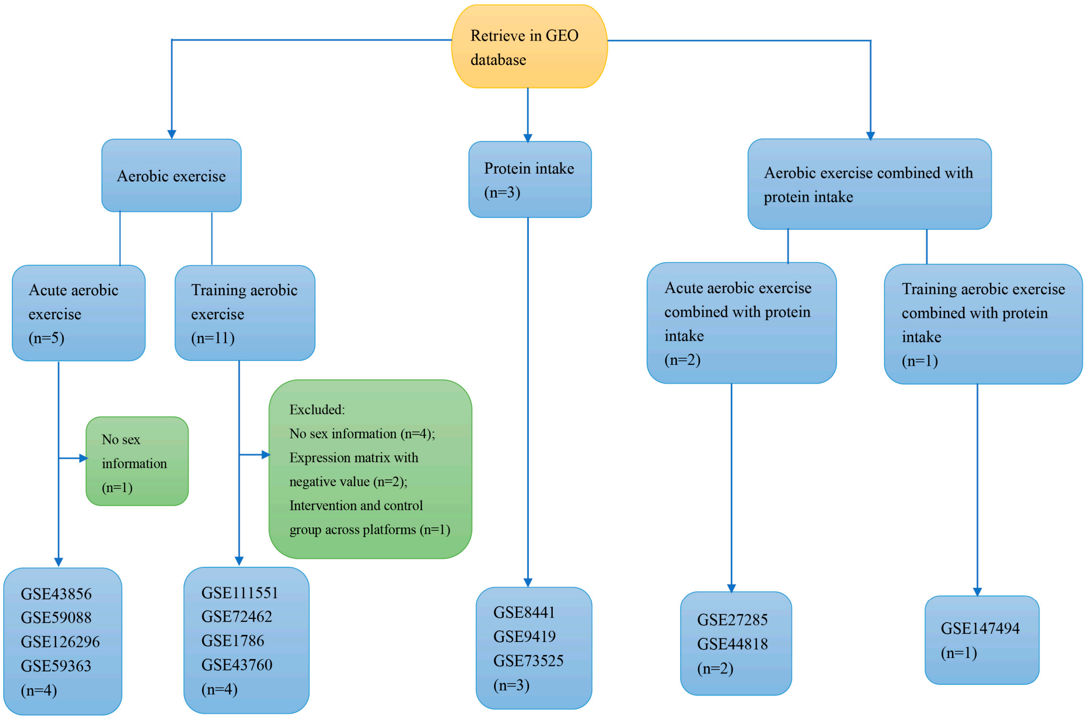

Supplementary Figure S1 Flowchart of the screening of GEO datasets.

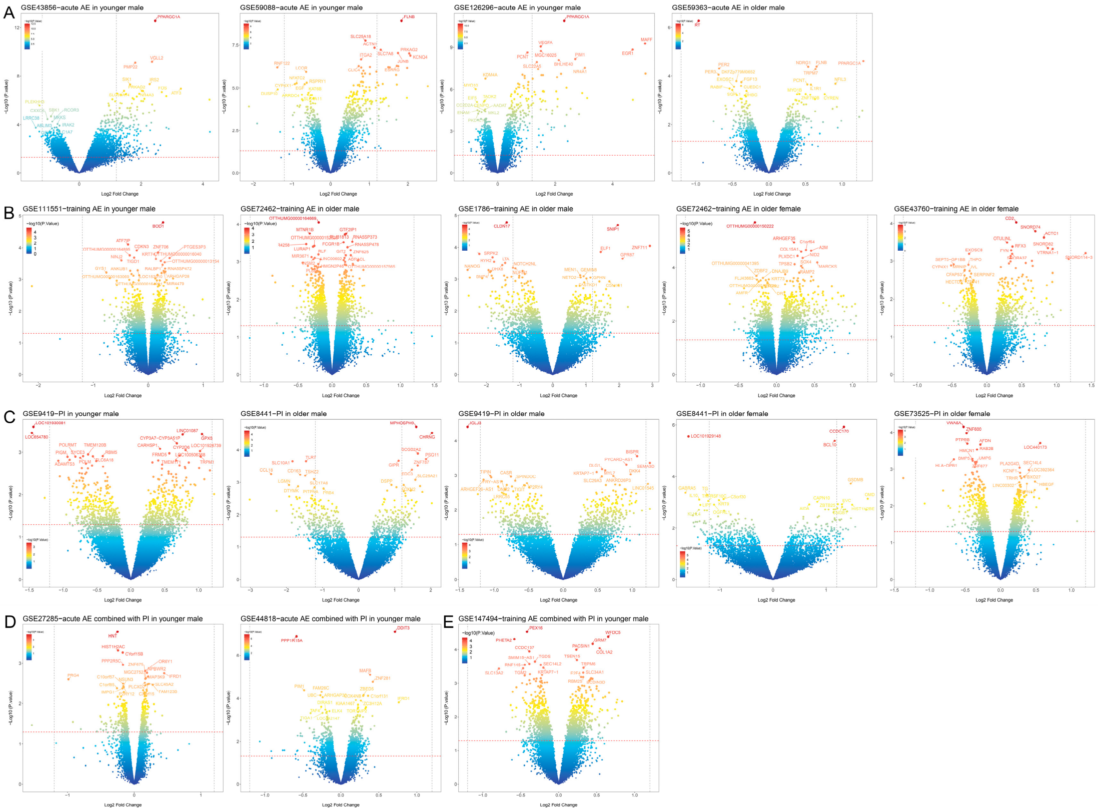

Supplementary Figure S2 Volcano plots across interventions for each dataset. The significantly upregulated and downregulated DEGs at the first 10 ranks were noted.

(A) Acute AE (GSE43856, GSE59088, GSE126296 and GSE59363); (B) Training AE (GSE111551, GSE72462, GSE1786 and GSE43760); (C) PI (GSE9419, GSE8441 and GSE73525); (D) Acute AE combined with PI (GSE27285 and GSE44818); (E) Training AE combined with PI (GSE147494).

Note: AE, aerobic exercise; PI, protein intake.

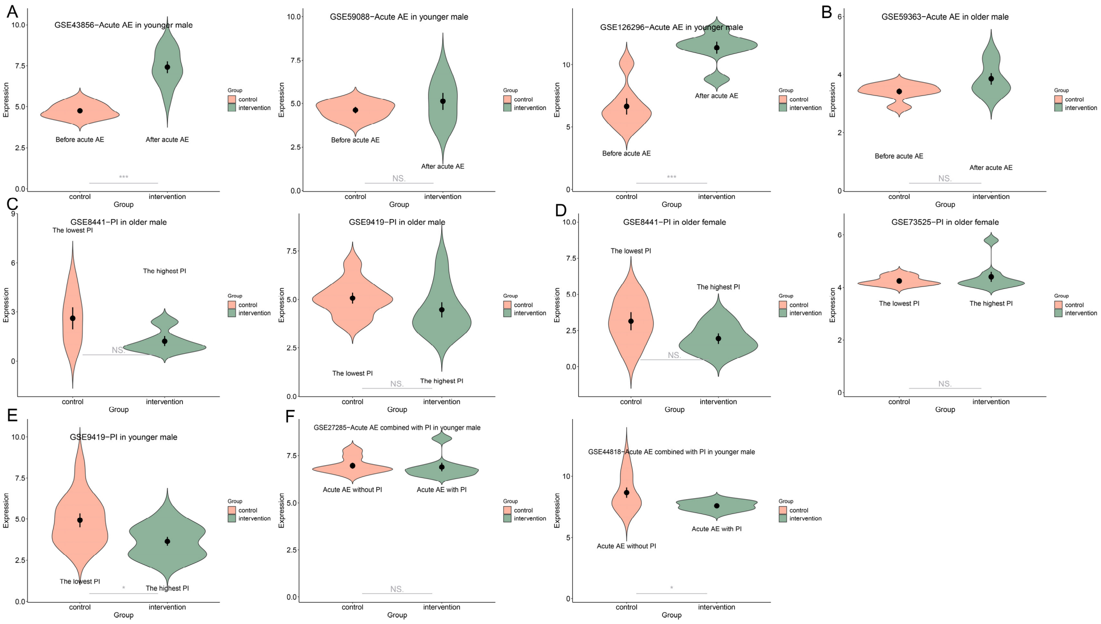

Supplementary Figure S3 (A-F) The expression levels of *FOS* in each dataset by different interventions. (A) Acute AE in the younger male (GSE43856, GSE59088 and GSE126296); (B) Acute AE in the older male (GSE59363); (C) PI in the older male (GSE8441 and GSE9419); (D) PI in the older female (GSE8441 and GSE73525); (E) PI in the younger male (GSE9419); (F) Acute AE combined with PI in the younger male (GSE27285 and GSE44818).

Note: AE, aerobic exercise; PI, protein intake.

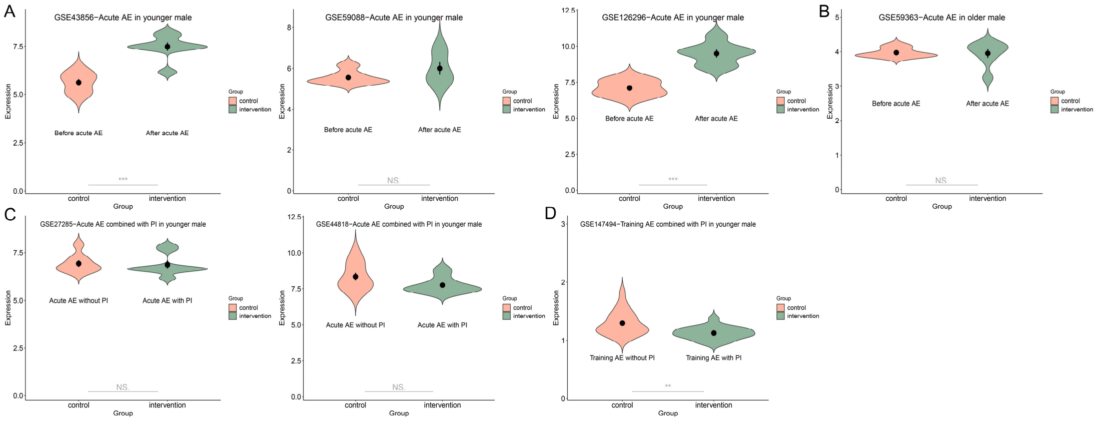

Supplementary Figure S4 (A-D) The expression levels of *MYC* in each dataset by different interventions. (A) Acute AE in the younger male (GSE43856, GSE59088 and GSE126296); (B) Acute AE in the older male (GSE59363); (C) Acute AE combined with PI in the younger male (GSE27285 and GSE44818); (D) Training AE combined with PI in the younger male (GSE147494).

Note: AE, aerobic exercise; PI, protein intake.

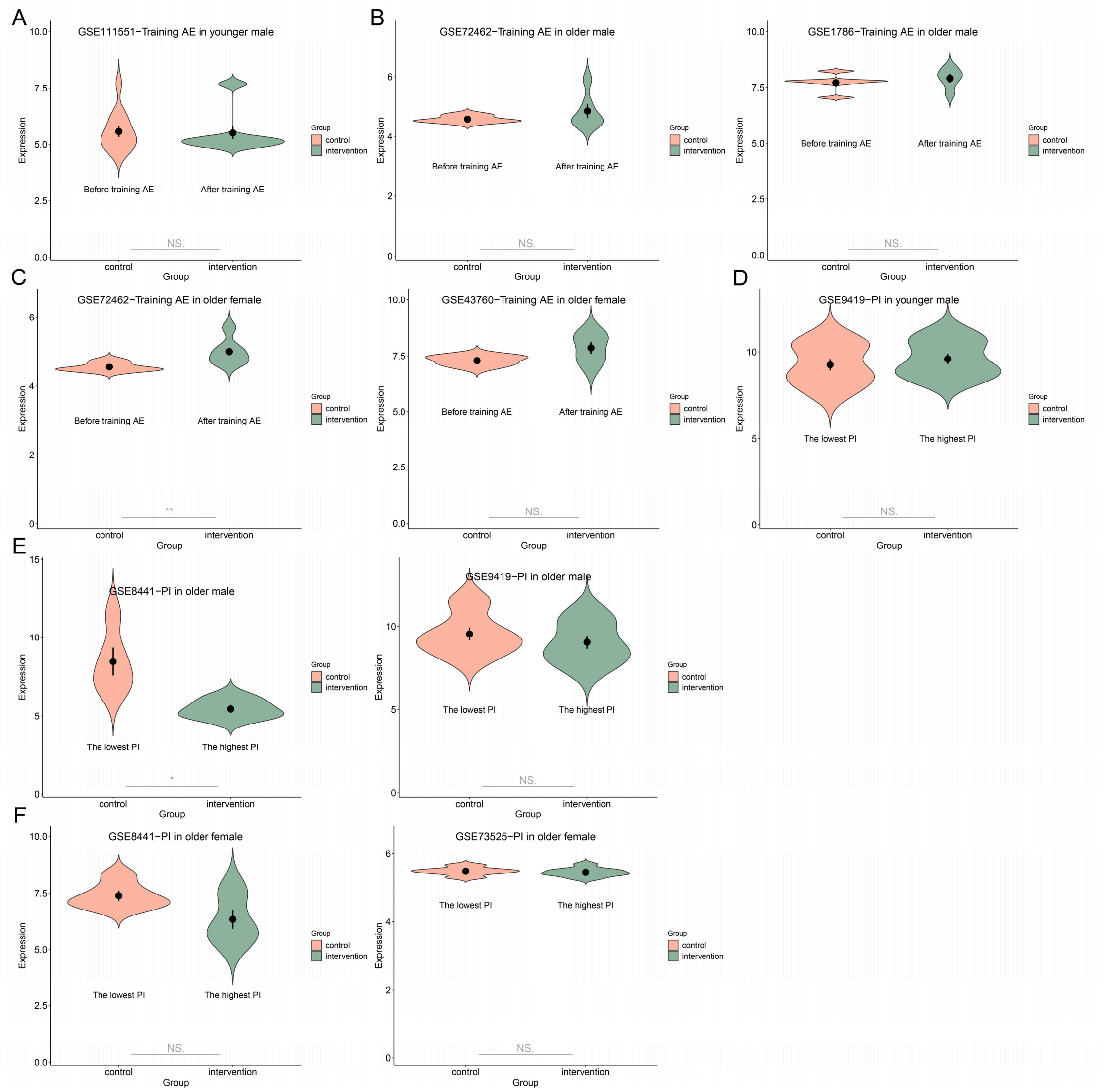

Supplementary Figure S5 (A-G) The expression levels of *COL1A1* in each dataset by different interventions. (A) Training AE in the younger male (GSE111551); (B) Training AE in the older male (GSE72462 and GSE1786); (C) Training AE in the older female (GSE72462 and GSE43760); (D) PI in the younger male (GSE9419); (E) PI in the older male (GSE8441 and GSE9419); (F) PI in the older female (GSE8441 and GSE73525).

Note: AE, aerobic exercise; PI, protein intake.

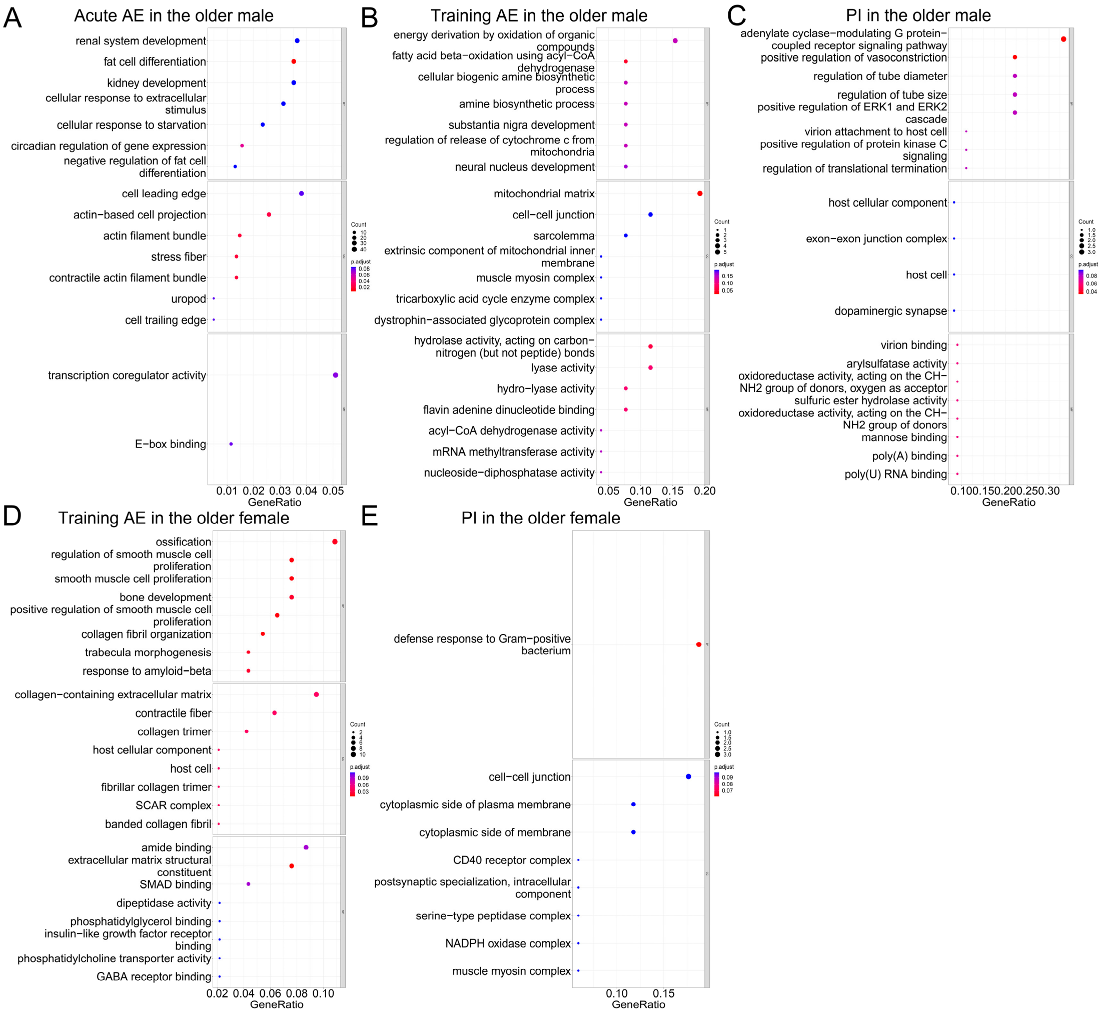

Supplementary Figure S6 The GO enrichment analysis of co-DEGs from varied interventions in the older people. (A) acute AE in the older male; (B) training AE in the older male; (C) PI in the older male; (D) Training AE in the older female; (E) PI in the older female.

Note: GO, Gene Ontology; co-DEGs, co- differentially expressed genes; AE, aerobic exercise; PI, protein intake.

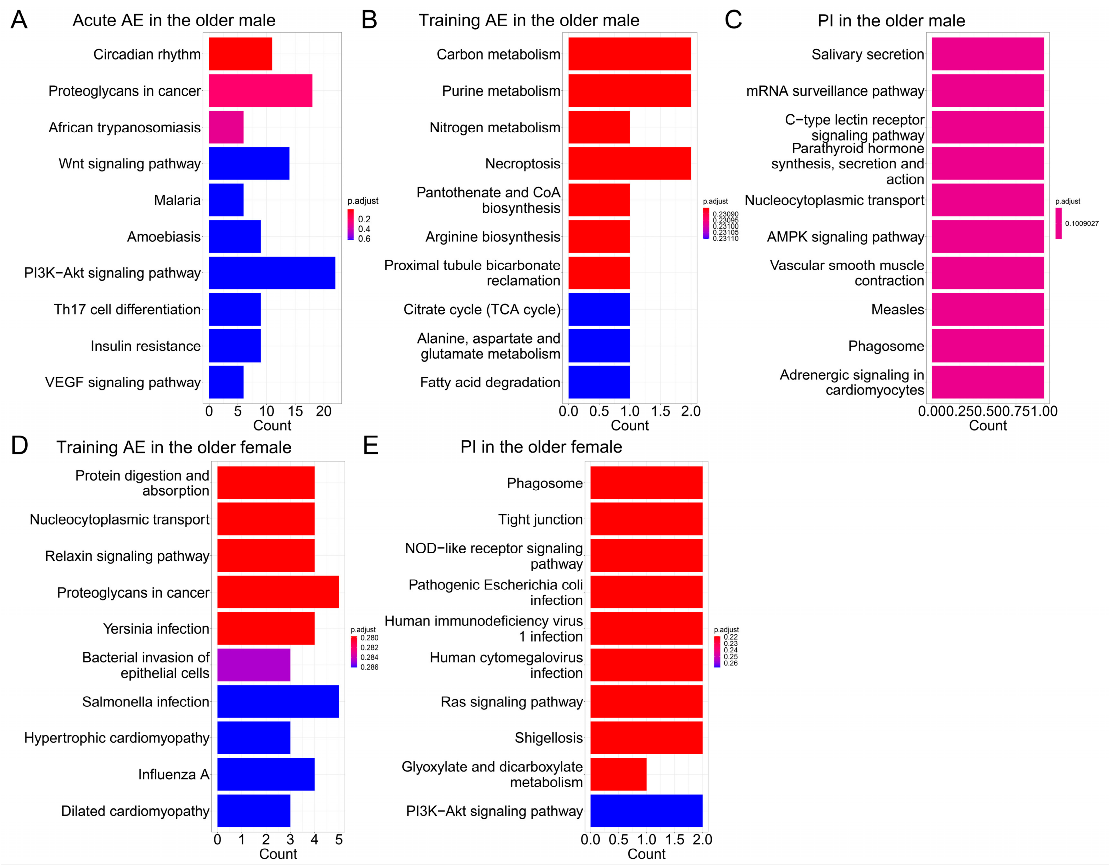

Supplementary Figure S7 The KEGG enrichment analysis of co-DEGs from varied interventions in the older people. (A) acute AE in the older male; (B) training AE in the older male; (C) PI in the older male; (D) Training AE in the older female; (E) PI in the older female.

Note: KEGG, Kyoto Encyclopedia of Genes and Genomes; co-DEGs, co-differentially expressed genes; AE, aerobic exercise; PI, protein intake.

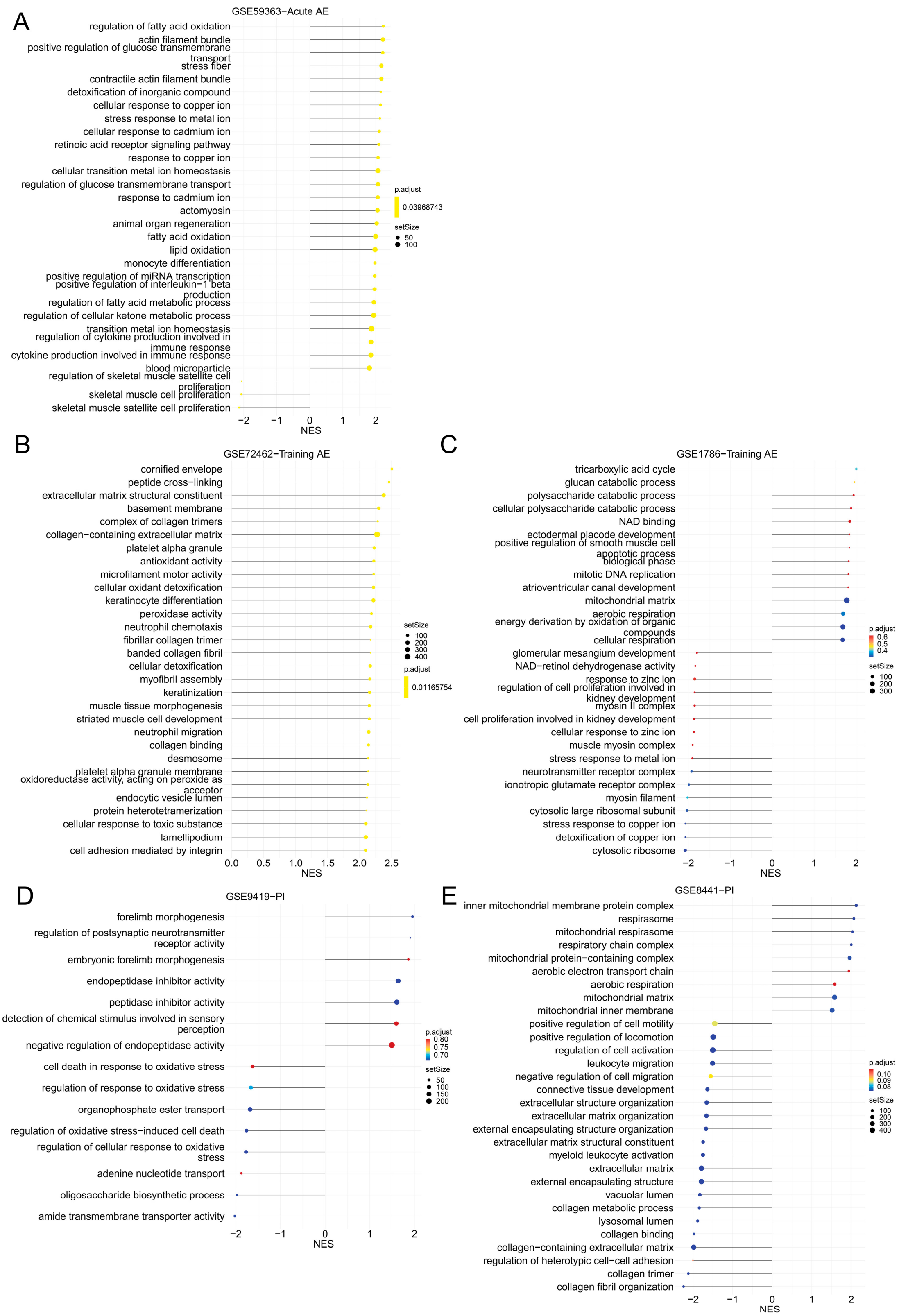

Supplementary Figure S8 The gene sets related GO terms from GSEA in each dataset by different interventions in the older male. (A) GSE59363-Acute AE; (B) GSE72462-Training AE; (C) GSE1786-Training AE; (D) GSE9419-PI; (E) GSE8441-PI.

Note: GO, Gene Ontology; GSEA, gene set enrichment analysis; AE, aerobic exercise; PI, protein intake; NES, normalized enrichment score.

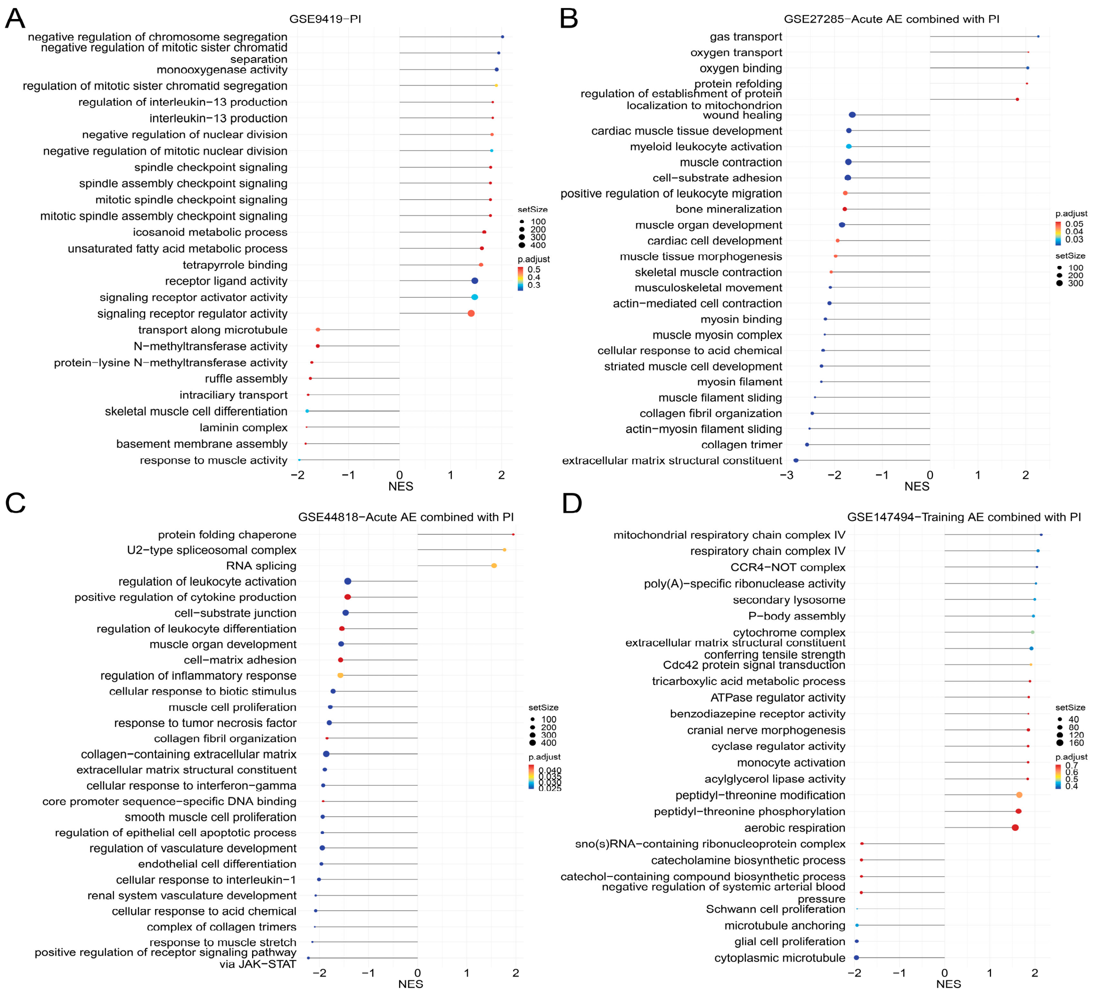

Supplementary Figure S9 The gene sets related GO terms from GSEA in each dataset by varied interventions in the younger male. (A) GSE9419-PI; (B) GSE27285-Acute AE combined with PI; (C) GSE44818-Acute AE combined with PI; (D) GSE147494-Training AE combined with PI.

Note: GO, Gene Ontology; GSEA, gene set enrichment analysis; AE, aerobic exercise; PI, protein intake; NES, normalized enrichment score.

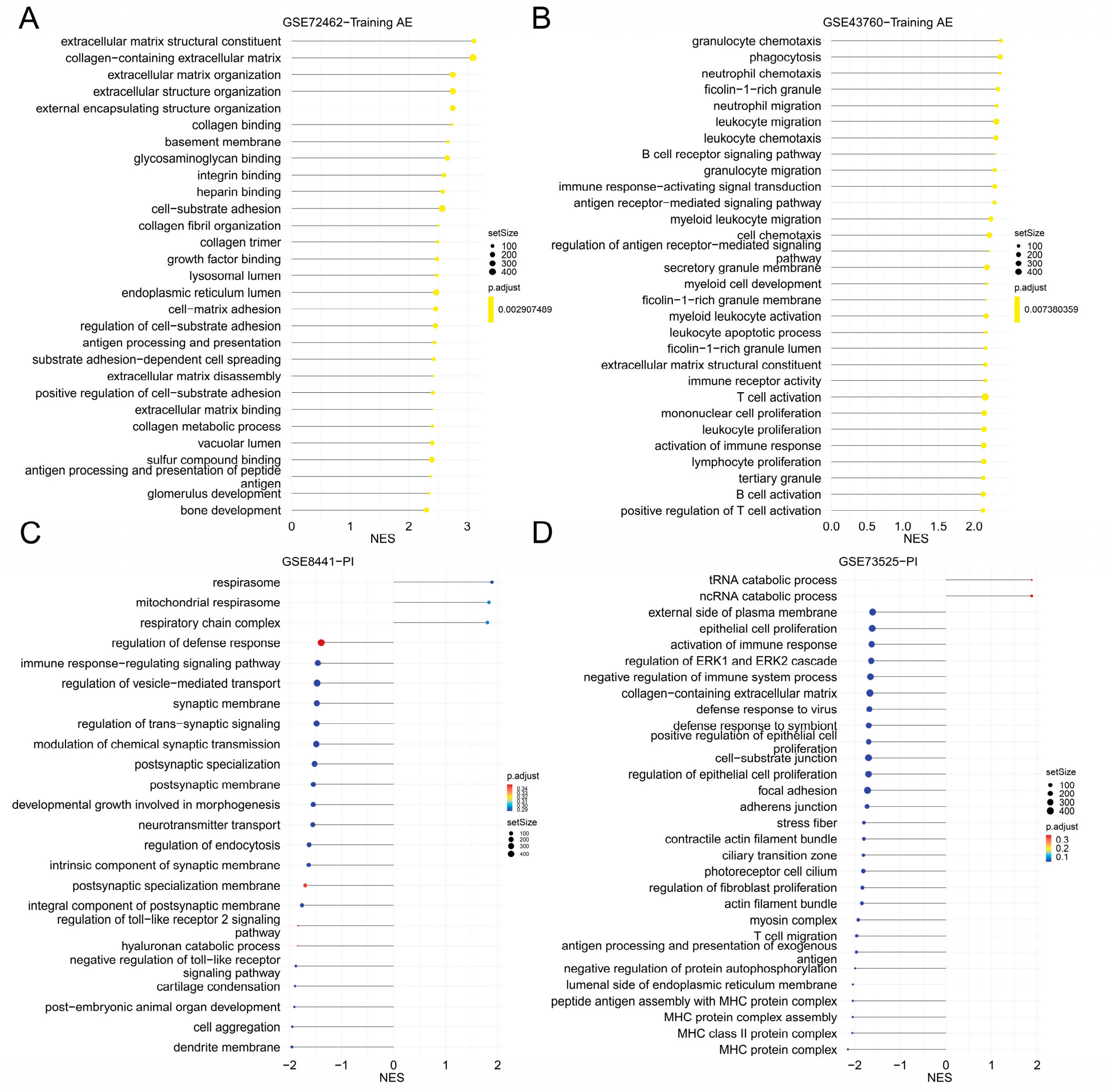

Supplementary Figure S10 The gene sets related GO terms from GSEA in each dataset by different interventions in the older female. (A) GSE72462-Training AE; (B) GSE43760-Training AE; (C) GSE8441-PI; (D) GSE73525-PI.

Note: GO, Gene Ontology; GSEA, gene set enrichment analysis; AE, aerobic exercise; PI, protein intake; NES, normalized enrichment score.

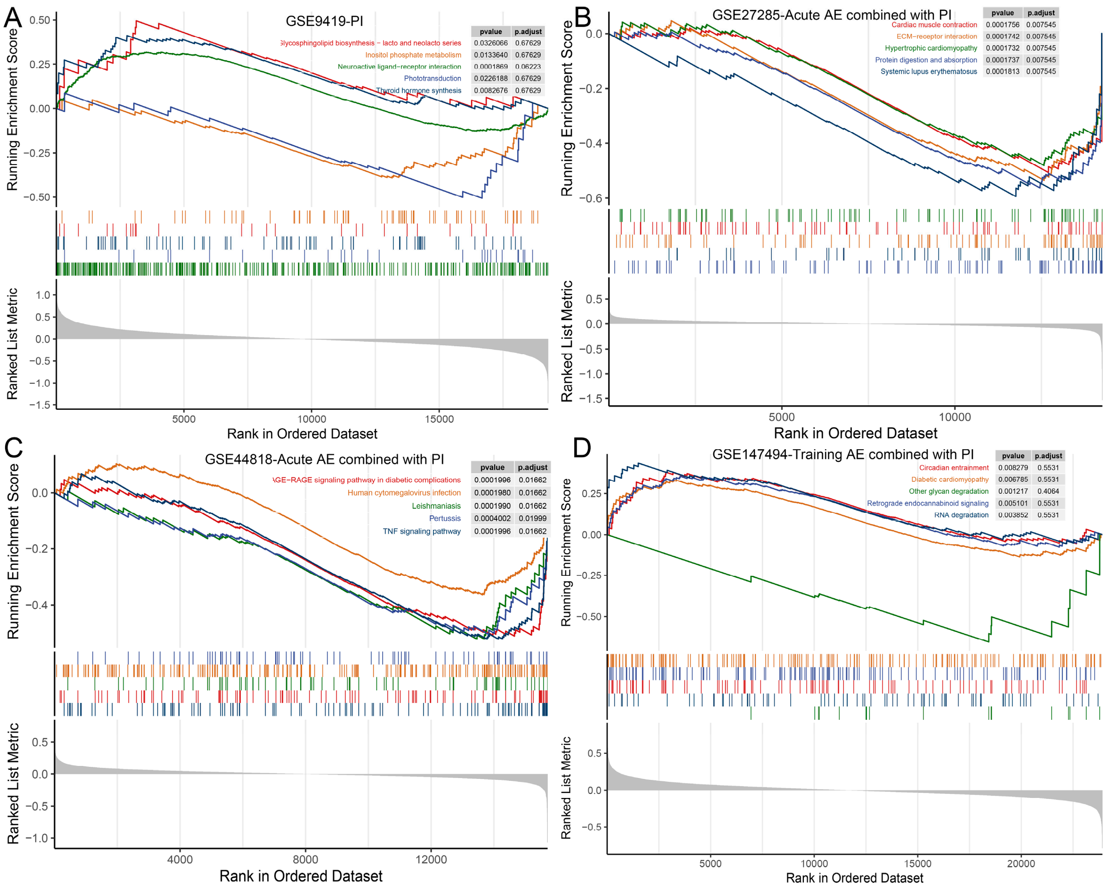

Supplementary Figure S11 Top 5 KEGG-pathway-related gene sets from GSEA by different interventions in the younger male. (A) GSE9419-PI; (B) GSE27285-Acute AE combined with PI; (C) GSE44818-Acute AE combined with PI; (D) GSE147494-Training AE combined with PI.

Note: KEGG, Kyoto Encyclopedia of Genes and Genomes; GSEA, gene set enrichment analysis; AE, aerobic exercise; PI, protein intake.

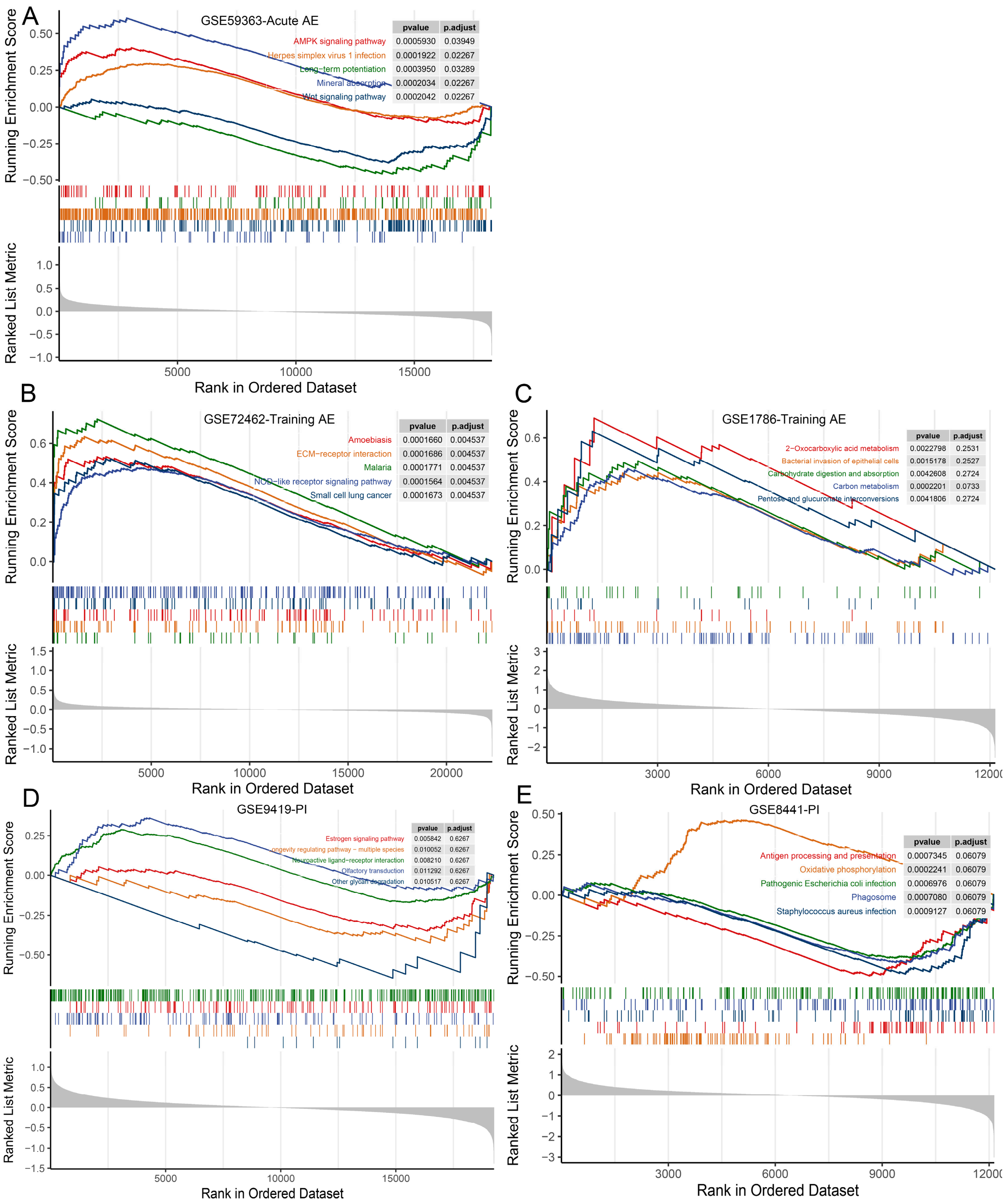

Supplementary Figure S12 Top 5 KEGG-pathway-related gene sets from GSEA in each dataset by different interventions in the older male. (A) GSE59363-Acute AE; (B) GSE72464-Training AE; (C) GSE1786-Training AE; (D) GSE9419-PI; (E) GSE8441-PI.

Note: KEGG, Kyoto Encyclopedia of Genes and Genomes; GSEA, gene set enrichment analysis; AE, aerobic exercise; PI, protein intake.

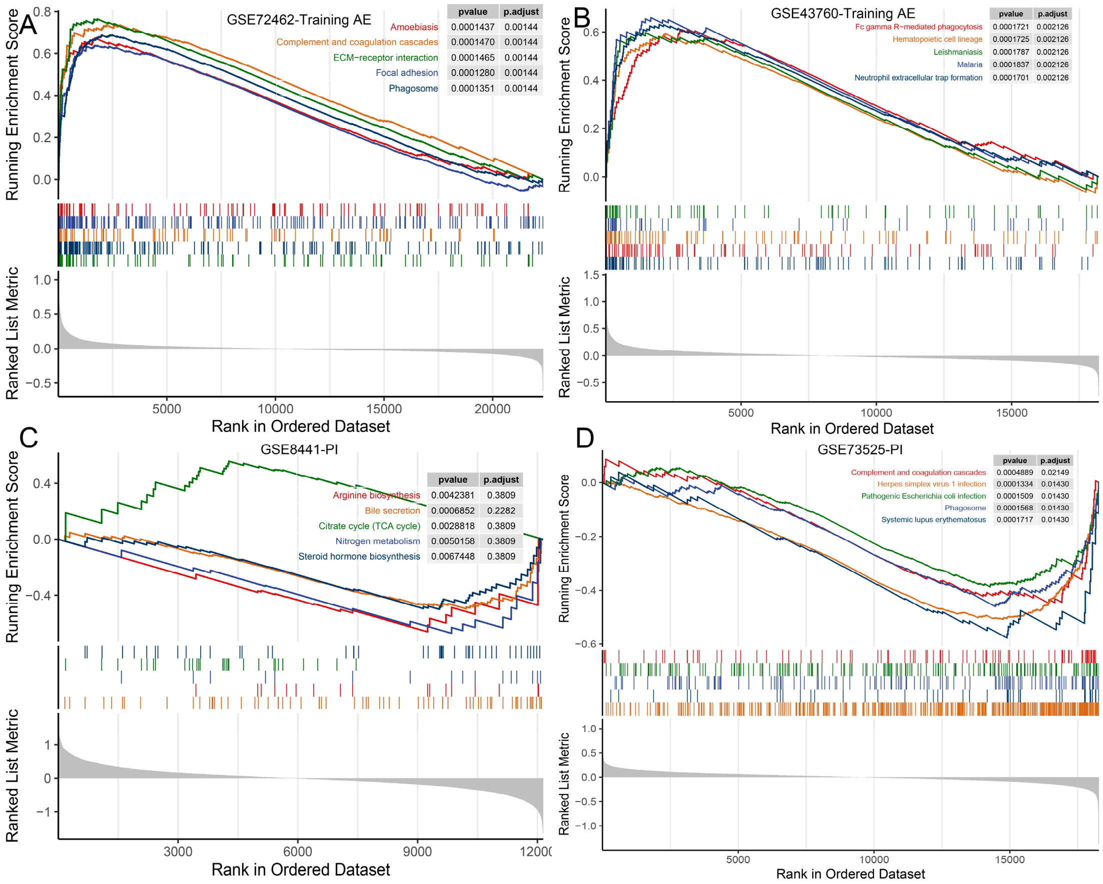

Supplementary Figure S13 The top 5 KEGG-pathway-related gene sets from GSEA in each dataset by different interventions in the older female. (A) GSE72462-Training AE; (B) GSE43760-Training AE; (C) GSE8441-PI; (D) GSE73525-PI.

Note: KEGG, Kyoto Encyclopedia of Genes and Genomes; GSEA, gene set enrichment analysis; AE, aerobic exercise; PI, protein intake.
